# Influencing Factors and Evaluation of the Self-Healing Behavior of Asphalt Binder Using Molecular Dynamics Simulation Method

**DOI:** 10.3390/molecules28062860

**Published:** 2023-03-22

**Authors:** Yan Li, Haiwei Zhang, Zirui Wu, Bowei Sun

**Affiliations:** 1 School of Civil Engineering and Architecture, Nanyang Normal University, Nanyang 473061, China; 2 School of Civil Engineering and Architecture, Zhengzhou University of Aeronautics, Zhengzhou 450046, China; 3 School of International Education, Henan University of Science and Technology, Luoyang 471000, China; 4 School of Transportation Science and Engineering, Civil Aviation University of China, Tianjin 300300, China

**Keywords:** asphalt binder, self-healing behavior, molecular dynamics simulation, oxidative aging, crack closing stage, intrinsic healing stage

## Abstract

In order to investigate the self-healing behavior of asphalt binder at the molecule scale, the self-healing models of neat and aged asphalt binder with different damage degrees were established by introducing a vacuum pad between two layers filled with asphalt molecules. With this model, the self-healing process was simulated at various healing conditions to reveal the effects of oxidative aging, damage degree and healing temperature on the self-healing property. In addition, self-healing efficiency was evaluated using the indexes representative of the characteristics of different self-healing stages. Our results show that the oxidative aging weakened the stacked structure of the asphalt binder and increased the healing activation energy barrier. The increasing damage degree extended the distance for particles to travel, thus prolonging the time required for the crack interfaces contacting with each other. The elevated temperature improved the molecular mobility by supplying more energy to the molecular system. Furthermore, the self-healing process was evaluated quantitatively by the density variation at the crack closing stage and the diffusion coefficient at the intrinsic healing stage. The duration of each stage was influenced by the oxidative aging, damage degree and healing temperature. The findings in this paper are helpful to reveal and evaluate the self-healing property of asphalt binder.

## 1. Introduction

Asphalt binder is a viscoelastic material that is very sensitive to temperature [1]. It has been found that when given a resting time at a suitable temperature, the cracks in asphalt binder can be healed automatically, together with the recovery of mechanical strength [2]. This phenomenon is called the intrinsic self-healing property of asphalt binder, which is conductive to improve asphalt pavement performance and extend the lifetime of asphalt pavement [3,4]. It has attracted close attention from researchers and become a hot research topic in recent years. Based on the previous research results, the self-healing behavior of asphalt binder is quite a complicated issue affected by various factors, such as aging degree [5,6], asphalt modifier [7], chemical structure [8], healing time and temperature [9,10], wax [11], etc. It has been widely accepted that the self-healing capability of asphalt binder is primarily determined by its chemical composition and molecular structure. The propagating and healing of cracks in asphalt binder all originate at the molecular scale. However, it is difficult to accurately reveal the relationship between the self-healing behavior and the molecular structure of asphalt binder only using traditional test methods.

With the rapid development of computer technology and chemical analysis technique, molecular dynamics (MD) simulations have been applied to explore the characteristics of asphalt materials at the molecular scale in the past years, which provides a new solution to the in-depth study of the self-healing behavior of asphalt binder. MD simulation is a computer virtual experiment on the molecular systems of materials, exhibiting benefits in terms of saving manpower and material resources [12]. It can be used to theoretically explain and predict the properties of materials based on Newtonian mechanics, providing strong support for the study of the structure–performance relationship. Up until now, MD simulation has been used in many research issues regarding asphalt materials, including asphalt modification [13], aged asphalt rejuvenation [14], asphalt–aggregate interface adhesion [15] and asphalt self-healing behavior [16].

In 2011, Bhasin et al. firstly introduced the MD method to the investigation of the self-healing behavior of asphalt binder [8]. They established an asphalt self-healing model by adding a vacuum pad between the layers filled with the asphalt molecular model, and found that the asphalt molecules with longer chain length and less chain branching had higher diffusivity across the crack interface. Sun et al. adopted MD simulations to evaluate the self-healing capacity of neat and SBS modified asphalt binders [17]. They also studied the effect of chemical composition and microstructure of asphalt binder on its self-healing ability [18]. Furthermore, they discussed the influence of temperature on the self-healing performance, proposing that the optimal healing temperature range was 40.3–48.7 °C [1]. Gao and Liu [19] revealed the self-healing mechanism of bio-oil recycled asphalt (BRA) using MD simulations and put forward that the self-healing process of BRA was dominated by viscous flow at higher temperatures and elastic recovery at lower temperatures. Yu et al. reported that the crack width had a significant influence on asphalt’s self-healing property, and further explained the AFM and SEM results using MD simulations [16]. Xu et al. found that the aging process was unfavorable to the self-healing process of asphalt binder [5]. He et al. investigated the self-healing property of different asphalt molecular models and proposed that volume compression and molecule stretching contributed to the crack healing process [20]. All these practices demonstrate that MD simulation provides an effective method for exploring the self-healing behavior of asphalt binder. Nevertheless, the self-healing behavior of asphalt binder is very complex, and its characterization and evaluation methods are still unclear. There is still a lack of a comprehensive analysis on the influencing factors and a reliable evaluation indicator of the self-healing process with full consideration of the characteristics of the self-healing process.

In this study, the self-healing models of neat and aged asphalt binder with different crack widths were established using Materials Studio 7.0 (MS7.0) software. Then, the self-healing behavior was simulated on the model under different temperatures. The effects of oxidative aging, damage degree and healing temperature on the self-healing process were analyzed using parameters such as density, radial distribution function and relative concentration. Finally, the self-healing efficiency was evaluated according to indexes representative of the characteristics of self-healing stages.

## 2. Molecular Model Construction and Evaluation Method of Self-Healing Behavior

### 2.1. Construction of Asphalt Molecular Model

**Figure 1:**
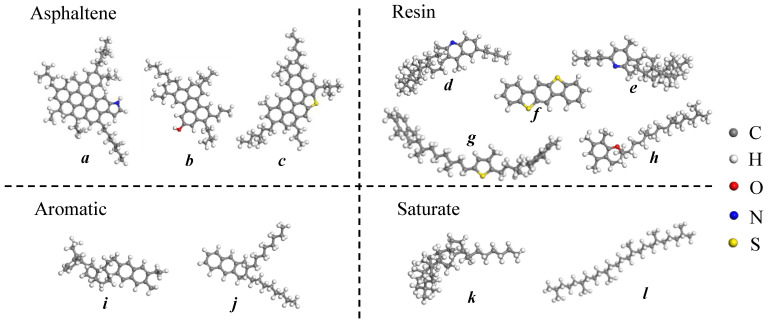
Representative molecules of the four components of neat asphalt binder: (**a**–**c**) asphaltene; (**d**–**h**) resin; (**i**,**j**) aromatic; (**k**,**l**) saturate.

Asphalt binder refined from crude oil consists of millions of molecules whose chemical composition and structure are quite complicated [15]. By using the chemical analysis method, the asphalt molecules are usually separated into three or four components. According to the four-component concept, asphalt binder is composed of asphaltene with the highest polarity, resin with the second polarity, aromatic with a weak polarity and saturate with non-polarity [5]. Asphaltene has the largest molecular weight and is the core of the asphalt colloid structure. Resin absorbs on the surface of asphaltene to form micelles that are then dissolved in the light components consisting of aromatic and saturate [21,22]. In this study, the classical four-component model of AAA-1 asphalt binder developed by Li and Greenfield is used [23], as shown in Figure 1.

**Figure 2:**
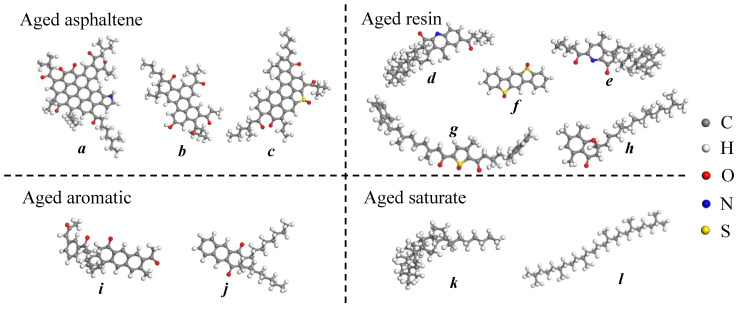
Representative molecules of the four components in aged asphalt binder: (**a**–**c**) aged asphaltene; (**d**–**h**) aged resin; (**i**,**j**) aged aromatic; (**k**,**l**) aged saturate.

The molecular model of aged asphalt binder was also constructed by introducing oxygen atoms at the possible oxidizable sites of the neat asphalt molecules, considering that the aging process of asphalt binder is dominated by the oxidation reaction. It has been discovered that the carbon and sulfur in asphalt binder are susceptible to oxidation, and their oxidation productions are ketone and sulfoxide, respectively [6,24]. Meanwhile, the benzylic carbon, defined as the first carbon connected with the aromatic ring, is also a readily oxidizable site. Hence, the oxygen atom can be introduced by replacing the hydrogen atom bonded to the benzylic carbon atom, resulting in the formation of ketones. As illustrated in Figure 2, the molecules of asphaltene, resin and aromatic are all added with oxygen atoms at the possible oxidizable sites to simulate the aging process [5]. The saturate is assumed to have the same molecular structure in the neat and aged asphalt binder because it is nonpolar and is difficult to oxidize.

**Figure 3:**
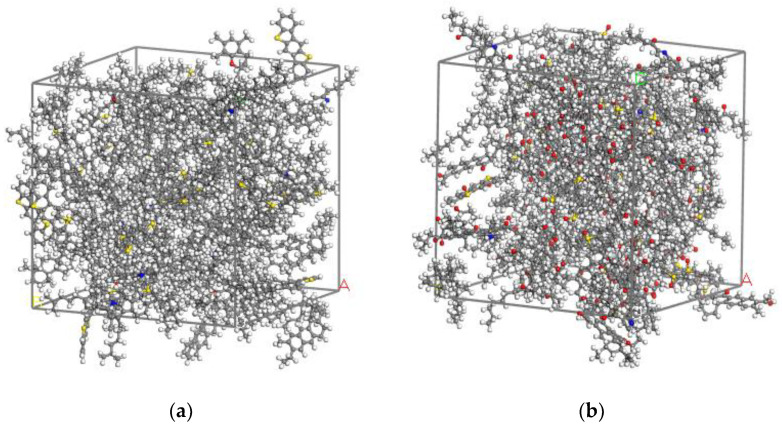
The optimized amorphous cell of asphalt binder: (**a**) neat asphalt binder; (**b**) aged asphalt binder.

The molecules with specified numbers (see Table 1) were put into a cubic box with an initial density of 0.5 g/cm^3^ to construct the amorphous cell of neat and aged asphalt binder with periodic boundary conditions using MS7.0 software. Then, geometry optimization was performed on the model to bring it to the lowest energy. Subsequently, the dynamics simulation was carried out under the canonical (NVT) ensemble to reach the targeted temperature and then under the isothermal–isobaric (NPT) ensemble at 1 standard atmosphere pressure for 100 ps to approach the real density of asphalt binder. Finally, a 100 ps dynamics simulation was performed again under the NVT ensemble to make the asphalt molecular model reach a state of greater equilibrium. The last frames of the neat and aged asphalt molecular models are depicted in Figure 3.

### 2.2. Verification of Asphalt Molecular Model

It is important to ensure that the asphalt molecular model is rational and applicable so that it can provide accurate simulation results. The thermodynamic parameters, including density and solubility, were used to verify the rationality of the asphalt molecular model. The density was simulated in the Forcite dynamics module under the NPT ensemble for 100 ps at different temperatures. The solubility simulations were performed in the Forcite cohesive energy density module to identify the stability of the asphalt molecular model. The simulated values of density and solubility are listed in Table 2. The simulated density is in good agreement with the density values reported previously. As expected, the density of aged asphalt binder is slightly higher than that of neat asphalt binder. The simulated solubility is also consistent with previous values, 15.3–23.0 (J/cm^3^)^0.5^ [16]. For the aged asphalt binder, the solubility parameter is increased to a certain extent, but still in the allowable range. In a word, the simulated values of density and solubility of neat and aged asphalt are all in the reasonable range. Thus, the asphalt molecular model is capable of reflecting the macro properties of normal asphalt and provided reliable results in the following simulations.

### 2.3. Development of Asphalt Self-Healing Model

**Figure 4:**
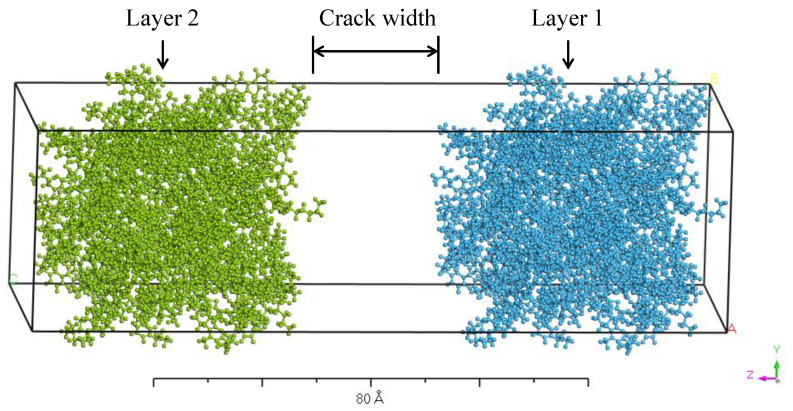
Self-healing model of asphalt binder.

The self-healing model of asphalt binder was established using the Build Layer module embedded in the MS7.0 software. It consisted of two layers and a vacuum pad between them, as shown in Figure 4. Layer 1 and layer 2 were filled with the same asphalt molecular model, and thus they were identical in chemical composition, molecular structure, etc. The crack was represented by the vacuum pad, whose width corresponded to the damage degree of the asphalt self-healing model. The self-healing process was simulated on the layered model under the NPT ensemble at 100 ps to investigate the crack closing process. Then, the layered model with a full closed crack was taken out as an example to analyze the molecular inter-diffusion and randomization across the closed crack interfaces under the NVT ensemble for 100 ps. The force field used in this simulation was the COMPASSⅡ (Condensed-phase Optimized Molecular Potentials for Atomistic Simulation Studies) force field, which is an extension and optimization of the COMPASS force field. COMPASSⅡ is applicable to a wide range of covalent molecules, including the most common organic compounds, small inorganic molecules and polymers. It provides a basis for accurately predicting the material properties of various chemicals.

### 2.4. Evaluation Method of Self-Healing Behavior

Based on the previous studies, the self-healing process of asphalt binder is divided into two stages in this paper, namely the crack closing stage (stage 1) and the intrinsic healing stage (stage 2) [9,25,26]. The self-healing behaviors at these two stages have their own characteristics and thus need to be evaluated using indicators that are representative of the behavior at each stage. In this paper, the density recovery ratio and the diffusion coefficient are selected for evaluating the self-healing efficiency of stage 1 and stage 2, respectively.

#### 2.4.1. Crack Closing Stage

At the crack closing stage, the self-healing efficiency is assessed using the crack closing rate. It is hard to accurately define and extract the crack width during the self-healing process. The density is an available parameter that can reflect the crack closing process. With the introduction of a crack, the initial density of the self-healing model falls below the normal value. When the crack interfaces come closer to each other, the density gradually recovers to the normal value. Hence, the self-healing efficiency is calculated using the variation in density, as illustrated in Equation (1).

(1) $$HE_{1}\left(t,T\right)=\frac{\rho \left(t,T\right)-\rho \left(t_{0},T\right)}{\rho \left(T\right)-\rho \left(t_{0},T\right)}$$

where *HE*_1_ is the self-healing efficiency of stage 1; $\rho \left(T\right)$ is the density of the optimized amorphous cell of the asphalt binder at temperature *T*; $\rho \left(t_{0},T\right)$ is the initial density of the self-healing model at temperature *T*; and $\rho \left(t,T\right)$ is the density of the self-healing model at simulation time *t*.

#### 2.4.2. Intrinsic Healing Stage

At the intrinsic healing stage, the diffusion coefficient is applied to judge the recovery ratio of mechanical strength of the asphalt binder. A higher diffusion coefficient means higher self-healing efficiency. The diffusion coefficient of particles can be calculated by the Einstein equation [27], as shown in Equations (2) and (3).

(2) $$D=\frac{1}{6}\underset{t\to \infty }{\lim}\frac{d}{dt}\overset{N}{\underset{i=1}{\sum }}{\left|r_{i}\left(t\right)-r_{i}\left(0\right)\right|}^{2}$$

(3) $$\mathrm{MSDt}={\left|r_{i}\left(t\right)-r_{i}\left(0\right)\right|}^{2}$$

where *D* is the diffusion coefficient of particles in the molecular system; *N* is the number of particles in the molecular system; MSD (mean square displacement) is the mean square of particle displacement compared with its initial position; $r_{i}\left(0\right)$ is the initial displacement of particle i; $r_{i}\left(t\right)$ is the displacement of particle i at time *t*; the angle bracket means the overall average of all particles during the simulation time.

By substituting Equation (3) in Equation (2), the diffusion coefficient *D* can be expressed as Equation (4).

(4) $$D=\frac{a}{6}$$

where a is the slope of the MSD-time curve.

Based on the Arrhenius theory, the logarithm of the diffusion coefficient *D* is derived as a linear function of the reciprocal of temperature, as expressed in Equation (5). It is introduced to verify the reliability of the simulation results in this study.

(5) $$\ln D=\ln D_{0}-\frac{Q}{R}\times \frac{1}{T}$$

where *D*_0_ is the diffusion constant; *Q* is the healing activation energy; *R* is the universal gas constant (8.314 J/mol/K); and *T* is the temperature (K).

## 3. Results and Discussion

### 3.1. Influencing Factors of Self-Healing Behavior

#### 3.1.1. Oxidative Aging

**Figure 5:**
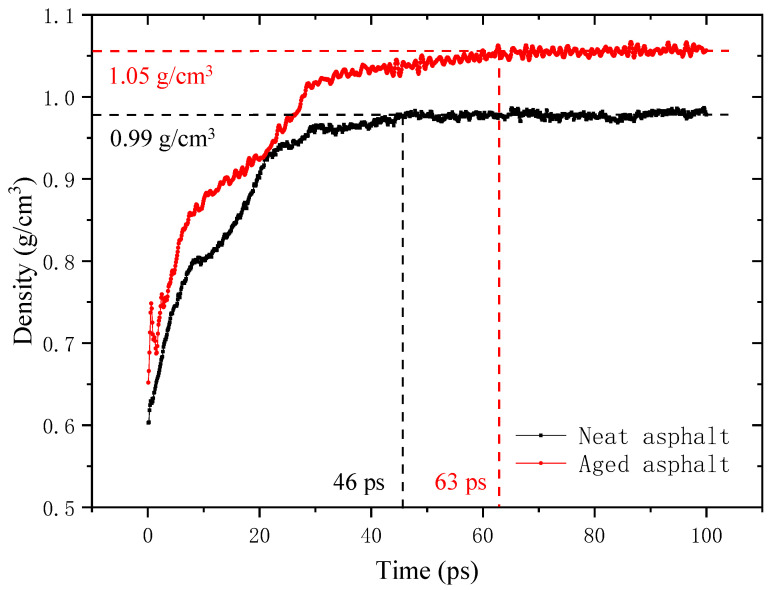
Density variation of the self-healing model of neat and aged asphalt binder with 1 nm crack at 298.15 K.

The density variation in the neat and aged asphalt binder with a 1 nm crack at 298.15 K during the crack closing process is depicted in Figure 5. The density recovery of the neat asphalt binder is completed at 46 ps, and it is extended to 63 ps for the aged asphalt binder, which marks the ending of the crack closing stage and the beginning of the intrinsic healing stage.

(1) At the crack closing stage, the density increases sharply with time until approaching the real density of undamaged asphalt binder, indicating that the crack is gradually closed. The oxidative aging reduces the growth ratio of the density and prolongs the simulation time required for density recovery;
(2) At the intrinsic healing stage, the asphalt molecules further diffuse across the closed crack interfaces to reach a greater equilibrium and randomization situation, contributing a lot to the strength recovery of the model. The density of the asphalt self-healing model stays at a stable value, and the aged asphalt binder has a higher density caused by the introduced oxygen atoms.

**Figure 6:**
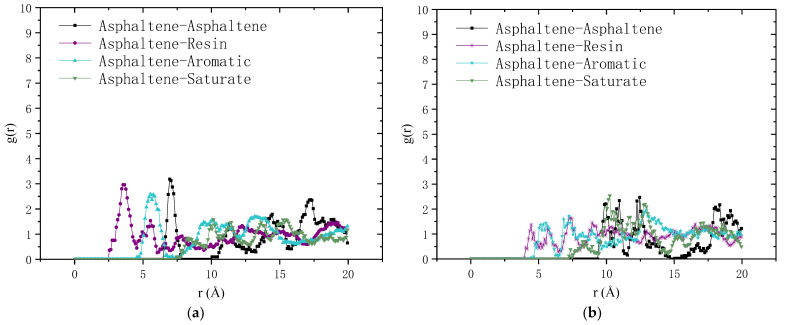
Radial distribution function for the pairs of asphaltene with the four components of asphalt binder: (**a**) neat asphalt binder; (**b**) aged asphalt binder.

The effect of oxidative aging can also be revealed by the change in molecular structure that can be described by the radial distribution function (RDF). In this study, the RDF is calculated using the mass center of asphalt molecules based on the trajectories of the optimized amorphous cell of the asphalt binder. RDF is defined as the normalized density distribution of a particle as a function of the distance (r) from the reference particle. It represents the probability distribution of a particle occurring around the reference particle. The value of RDF equals 1 when the particle is uniformly distributed around the reference particle without aggregation. Figure 6 shows the RDFs of an asphaltene–asphaltene pair, asphaltene–resin pair, asphaltene–aromatic pair and asphaltene–saturate pair. It can be seen that the RDF of the asphaltene–asphaltene pair of neat asphalt binder has a distinct first peak value at the distance of 6–8 Å and a second weaker peak value at 14–18 Å. This indicates that the asphaltene tends to aggregate at the first peak position to form a stacked structure that is gradually disappeared with the increasing distance. Both the asphaltene–resin pair and the asphaltene–aromatic pair have first peaks occurring at a shorter distance compared with the first peak of the asphaltene–asphaltene pair. This might be attributed to the fact that resin and aromatic are small molecules serving as the dispersion medium of asphaltene. The RDF first peak of the asphaltene–saturate pair has a relatively lower value and longer distance compared with other pairs in neat asphalt binder, demonstrating that the saturate is incompatible with the asphaltene. In the aged asphalt binder, the first peak of RDF of the asphaltene–asphaltene pair has a smaller value and a longer distance, suggesting that aging decreases the aggregation phenomenon of asphaltene and weakens the stacked structure. Meanwhile, oxidative aging also decreases the RDF peak value of the pairs of asphaltene with resin and saturate, while it has little influence on the RDF of the asphaltene–saturate pair. In general, oxidative aging has obvious influence on the molecular structure of asphalt binder, pulling the molecules apart from the core of asphalt collision and changing the alignment of the chemical components.

#### 3.1.2. Damage Degree

**Figure 7:**
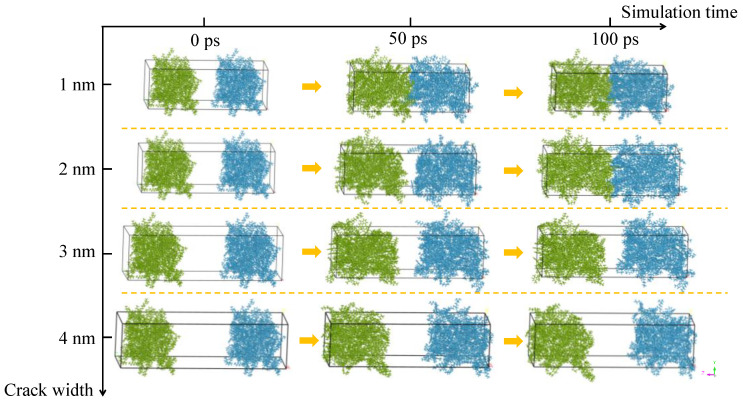
Snapshots of asphalt self-healing model with crack width of 1–4 nm.

The damage degree is represented by the crack width in the self-healing model. Larger crack width means higher damage degree. The snapshots of self-healing models with different crack widths during the crack closing process at 298.15 K are illustrated in Figure 7. During the self-healing process, the asphalt molecules on the two sides of the crack gradually diffuse into the crack and entangle with each other, contributing to the narrowed crack width. With the increasing in crack width, the difficulty in closing the crack is increased due to the extended distance for particles to travel and the weakened interaction between asphalt molecules on the crack interfaces. Generally speaking, the more serious the damage degree is, the more difficult to bring the crack interfaces back into contact with each other, and the larger the residual crack width is.

**Figure 8:**
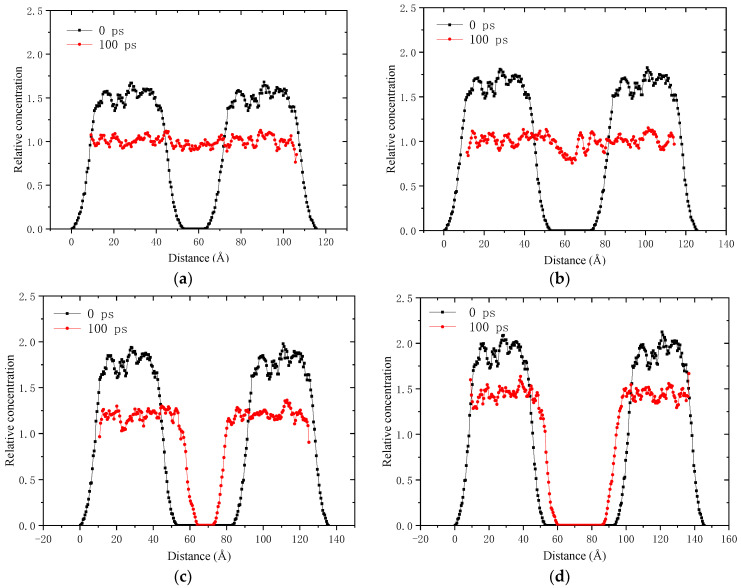
Relative concentration distribution curve along the z axis of the self-healing model of aged asphalt binder with 1–4 nm crack: (**a**) 1 nm; (**b**) 2 nm; (**c**) 3 nm; (**d**) 4 nm.

The relative concentration distribution of aged asphalt binder along the z axis is shown in Figure 8. It can be found that at the starting point of the simulation, the molecular distribution is a bimodal shape, with higher concentration at the peak and lower concentration at the edge. There are no molecules in the middle of the model, which is a vacuum space representing the crack. Under the action of density gradient, the atoms tend to move towards the crack and reach a more uniform distribution along the z axis. A healing time of 100 ps is sufficient for the closure of the 1 nm crack in the aged asphalt binder, but it needs to be prolonged for closing the 2–4 nm cracks.

#### 3.1.3. Healing Temperature

**Figure 9:**
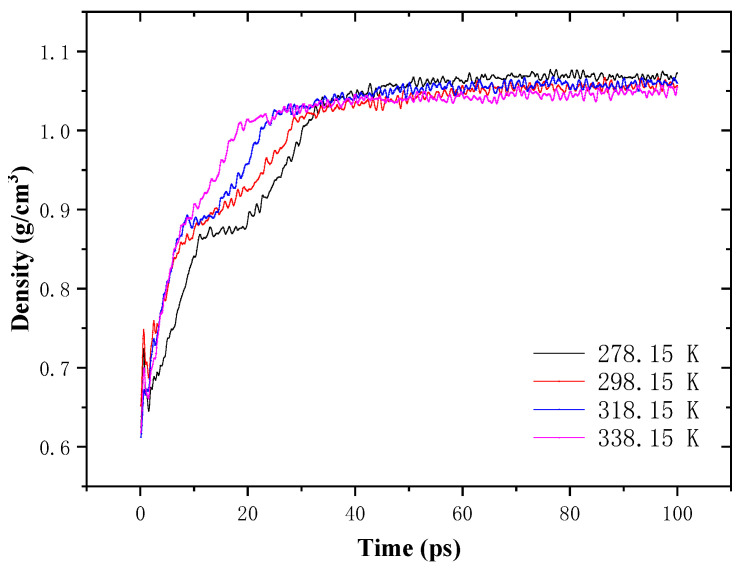
Density variation of the self-healing model of aged asphalt binder with 1 nm crack.

The density variation of aged asphalt binder containing a 1 nm crack at different temperatures is shown in Figure 9. The initial density is lower than the normal density of asphalt binder due to the introduction of the crack. In the first 10 ps, the system is heated to reach the target temperature, and thus the density is unstable. At the crack closing stage, raising the healing temperature results in an improved density recovery rate and a shortened duration time. At the intrinsic healing stage, the density keeps at a stable value that is affected by the healing temperature. It shows a inverse relationship with the rising temperature, which conforms to general knowledge. According to previous studies [26], strength recovery mainly happens at this stage and is highly dependent on time.

**Figure 10:**
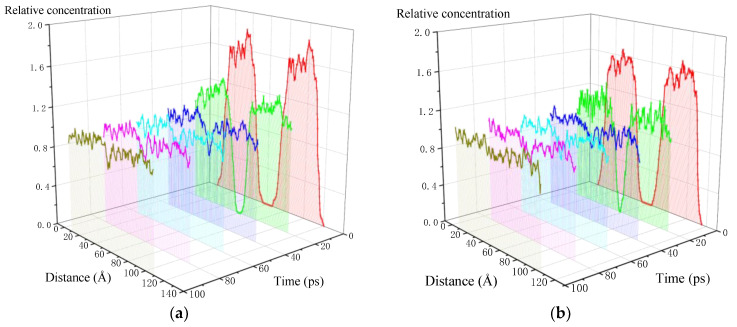

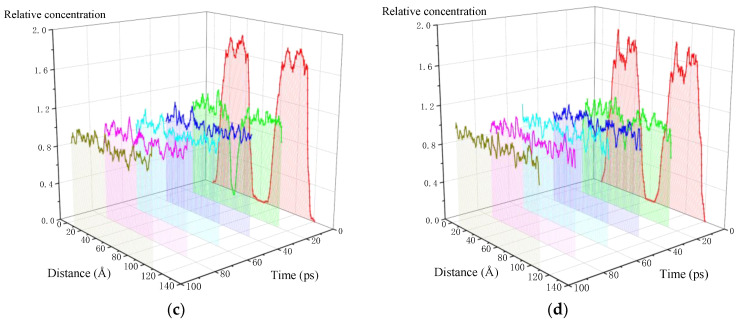
Relative concentration distribution curve along the z axis of aged asphalt binder with 1 nm crack: (**a**) 278.15 K; (**b**) 298.15 K; (**c**) 318.15 K; (**d**) 338.15 K.

In order to intuitively display the effect of healing temperature and time, the relative concentration profiles of the model along the z axis at different temperatures are depicted in Figure 10. With the extending of simulation time, the relative concentration along the z axis gradually reaches a uniform value, indicating that the crack is filled by the asphalt molecules. On the other hand, the self-healing efficiency is obviously enhanced by the elevated healing temperature. For example, at the simulation time of 20 ps, the crack still is still very obvious at 278.15 K, and it is narrowed at 298.15 K and 318.15 K. When the temperature is increased to 338.15 K, it is difficult to find the existence of crack at 20 ps.

### 3.2. Evaluation of Self-Healing Efficiency

#### 3.2.1. Crack Closing Stage

**Figure 11:**
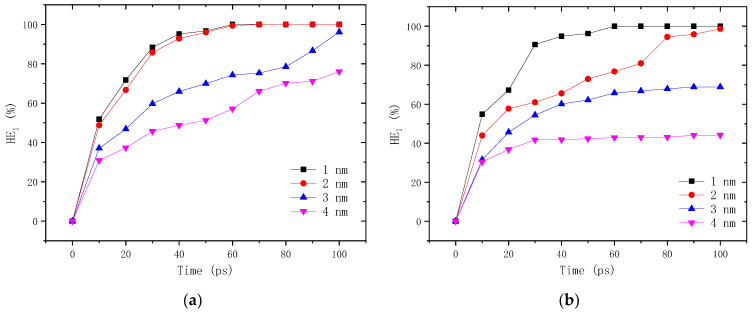
Self-healing efficiency of asphalt binder with different damage degrees at 298.15 K: (**a**) neat asphalt binder; (**b**) aged asphalt binder.

**Figure 12:**
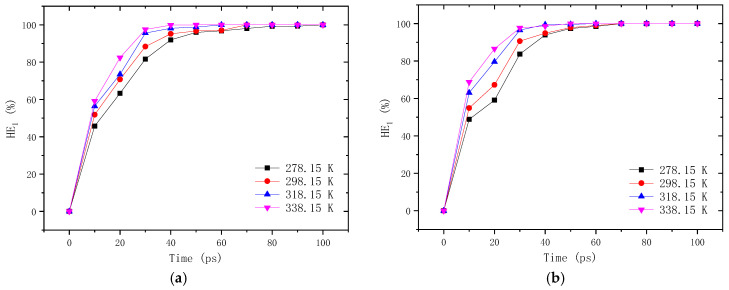
Self-healing efficiency of asphalt binder with 1 nm crack at different temperatures: (**a**) neat asphalt binder; (**b**) aged asphalt binder.

At the crack closing stage, the density gradient is the main driving force for the self-healing process due to the existence of crack, and thus the characteristics of this stage can be described by the variation of density with time. According to Equation (1), the self-healing efficiency of neat and aged asphalt self-healing models with different crack widths are calculated and depicted in Figure 11. It can be found that the self-healing efficiency increases consistently with time, but the growth ratio gradually slows down. With the increasing crack width, the self-healing efficiency is reduced significantly, leading to the longer time consumed to close the cracks. For example, the self-healing efficiency of the neat asphalt model with a 1–2 nm crack reaches 100% at 60 ps for the neat asphalt binder, while the cracks larger than 2 nm are not fully closed even after a simulation time of 100 ps. What is more, the aging of asphalt binder decreases the self-healing efficiency greatly, especially for the model with cracks of 2–4 nm. This might be attributed to the increased molecular weight and the weakened mobility of the asphalt molecules, as explained in Section 3.1.1. The healing temperature also has a certain influence on the crack closing rate, as shown in Figure 12. In the temperature range of 278.15–338.15 K, the higher the temperature is, the higher the self-healing efficiency is, and the shorter the time consumed to fully close the crack. Nevertheless, it shows a weaker influence on the density healing efficiency compared with the damage degree.

#### 3.2.2. Intrinsic Healing Stage

**Figure 13:**
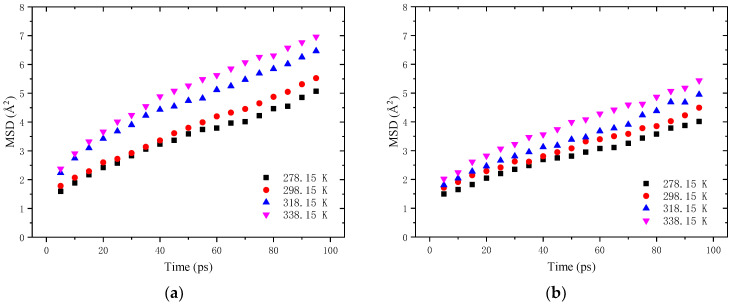
MSD-time curve of self-healing model of asphalt binder with 1 nm crack at the intrinsic healing stage: (**a**) neat asphalt binder; (**b**) aged asphalt binder.

After the crack is fully closed (*HE*_1_ = 100%), the self-healing process enters into the second stage, namely the intrinsic healing stage. As can be found in Figure 11 and Figure 12, the starting time of the second stage varies with the asphalt type, damage degree and healing temperature. At this stage, the density value is relatively stable, and the self-healing behavior is dominated by the inter-diffusion and randomization of asphalt molecules across the closed crack interfaces. Figure 13 illustrates the MSD curve of neat and aged asphalt self-healing models with a 1 nm crack at the intrinsic healing stage. It can be seen that the curve increases linearly with simulation time and temperature. This can be explained by the fact that more energy is transferred to the asphalt molecular system at a higher temperature, making it easier for the molecules to climb the activation energy barrier and diffuse across the crack interfaces [28]. In addition, compared with the neat asphalt, the MSD of aged asphalt is greatly reduced and is less susceptible to temperature.

The diffusion coefficient is calculated using Equation (4) based on the fitting curve of MSD data, as listed in Table 3. The diffusion coefficient increases stably with temperature, indicating that the molecular mobility is enhanced when more energy is supplied at a higher temperature. The aged asphalt molecules have a lower diffusion coefficient due to the increased molecular weight. Based on the analysis above, it can be deduced that the recovery of mechanical strength of aged asphalt is slower than that of neat asphalt, but it can be promoted by the elevated temperature.

**Figure 14:**
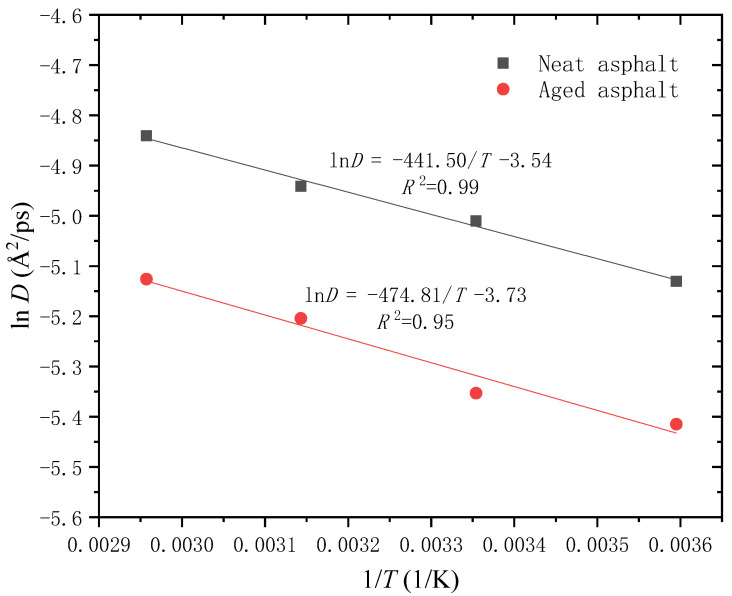
Relationship between ln*D* and 1/*T* of neat and aged asphalt binder at the intrinsic healing stage.

According to the data in Table 3, the variation of ln*D* with 1/*T* is obtained for the neat and aged asphalt, as plotted in Figure 14. It can be found that ln*D* has a good linear correlation with 1/*T*, which is in good agreement with Equation (5). This demonstrates that the simulation results are consistent with the theoretical analysis. Based on the fitting formula, the healing activation energy of the neat asphalt is calculated as 3.67 kJ/mol, which is a little lower than that of the aged asphalt (3.95 kJ/mol). The healing activation energy is the minimum energy needed for molecular diffusion and randomization, which can be adopted to rank the self-healing capacity of asphalt binders [28]. A lower value of healing activation energy means less energy is required for the self-healing process to initiate. Thus, it can be inferred that the neat asphalt binder has greater potential to recover mechanical strength at the intrinsic healing stage compared with the aged asphalt binder.

## 4. Conclusions

The self-healing behavior of asphalt binder has promising applications in improving the performance and lifetime of asphalt pavement. In this paper, the influencing factors and evaluation of the asphalt self-healing process were investigated using a molecular dynamics simulation method. The self-healing models of neat and aged asphalt binder were developed by introducing a vacuum pad between two layers filled with asphalt molecular models. The damage degree was simulated using the width of the vacuum pad that represented the crack. It is demonstrated that oxidative aging reduced the growth ratio of asphalt density and prolonged the simulation time required for density recovery. The increasing damage degree delayed the crack closing process. The more serious the damage degree was, the larger the residual crack width was, and the longer the healing time needed for completely closing the crack. Increases in healing temperature accelerated the self-healing process by supplying more energy to the asphalt molecular system. The self-healing process of the crack was divided into the crack closing stage (stage 1) and the intrinsic healing stage (stage 2). The duration of each stage varied with the aging condition, damage degree and healing temperature. The self-healing efficiency of asphalt binder was evaluated using density variation at stage 1 and the diffusion coefficient at stage 2. Oxidative aging had a negative effect on the self-healing efficiency, while extended healing time and elevated healing temperature were beneficial to the improvement of self-healing efficiency. Meanwhile, increasing crack width greatly reduced the self-healing efficiency, demonstrating a higher influence degree than the healing temperature.

## Figures and Tables

**Table 1:** Chemical components in the amorphous cell of asphalt binder.

Chemical Component	Code	Chemical Formula	Number
Asphaltene	a	C_66_H_81_N	2
	b	C_42_H_54_O	3
	c	C_51_H_62_S	3
Resin	d	C_40_H_59_N	4
	e	C_36_H_57_N	4
	f	C_18_H_10_S_2_	15
	g	C_40_H_60_S	4
	h	C_29_H_50_O	5
Aromatic	i	C_35_H_44_	11
	j	C_30_H_46_	13
Saturate	k	C_35_H_62_	4
	l	C_30_H_62_	4

**Table 2:** Density and solubility parameters of neat and aged asphalt binder.

Parameter	Aging Condition	278.15 K	298.15 K	318.15 K	338.15 K	Measured Value
Density (g/cm^3^)	Unaged	1.005	0.992	0.989	0.965	0.96–1.04 [16]
	Aged	1.060	1.051	1.046	1.037	
Solubility parameter (J/cm^3^)^0.5^	Unaged	18.11	17.84	17.78	17.19	15.3–23.0 [16]
	Aged	19.37	19.13	18.85	18.70	

**Table 3:** Fitting equation and diffusion coefficient of neat/aged asphalt binder.

Temperature	Neat Asphalt Binder			Aged Asphalt Binder		
	Fitting Formula	*R* ^2^	*D* (Å^2^/ps)	Fitting Formula	*R* ^2^	*D* (Å^2^/ps)
278.15 K	y = 0.0355x + 1.6673	0.99	0.0059	y = 0.0267 x + 1.4740	0.99	0.0045
298.15 K	y = 0.0400x + 1.7195	0.99	0.0067	y = 0.0284x + 1.6704	0.99	0.0047
318.15 K	y = 0.0429x + 2.48699	0.98	0.0072	y = 0.0330x + 1.7441	0.99	0.0055
338.15 K	y = 0.0474x + 2.6928	0.97	0.0079	y = 0.0356x + 2.0758	0.99	0.0059

## Data Availability

The research data are available by contacting the corresponding author.
